# Addressees Are Sensitive to the Presence of Gesture When Tracking a Single Referent in Discourse

**DOI:** 10.3389/fpsyg.2019.01775

**Published:** 2019-08-13

**Authors:** Sandra Debreslioska, Joost van de Weijer, Marianne Gullberg

**Affiliations:** ^1^Centre for Languages and Literature, Lund University, Lund, Sweden; ^2^Lund University Humanities Lab, Lund University, Lund, Sweden

**Keywords:** bimodal reference, gesture, discourse, speech-gesture relationship, anaphoric gesture, gesture perception, localizing gesture

## Abstract

Production studies show that anaphoric reference is bimodal. Speakers can introduce a referent in speech by also using a localizing gesture, assigning a specific locus in space to it. Referring back to that referent, speakers then often accompany a spoken anaphor with a localizing anaphoric gesture (i.e., indicating the same locus). Speakers thus create visual anaphoricity in parallel to the anaphoric process in speech. In the current perception study, we examine whether addressees are sensitive to localizing anaphoric gestures and specifically to the (mis)match between recurrent use of space and spoken anaphora. The results of two reaction time experiments show that, when a single referent is gesturally tracked, addressees are sensitive to the presence of localizing gestures, but not to their spatial congruence. Addressees thus seem to integrate gestural information when processing bimodal anaphora, but their use of locational information in gestures is not obligatory in every discourse context.

## Introduction

Discourse needs to be cohesive for addressees to understand it. They have to know at all times who is doing what to whom. Thus, speakers need to manage reference to discourse entities constantly and consistently, a process known as anaphoric reference. Most entities are mentioned multiple times throughout discourse. When a speaker mentions a referent for the first time, she will typically use a rich referential expression (e.g., an indefinite lexical noun phrase, “a woman”). Once the referent is introduced, the speaker has a choice of different anaphoric expressions with which to refer back to it. Depending on the referential context, that is whether the referent is maintained from one clause to the next or reintroduced after a gap, the speaker will either choose a lean expression (e.g., a pronoun, “she”) or a rich one (e.g., a definite lexical noun phrase, “the woman”), respectively. This is how speakers create cohesion in speech (Givón, 1983; Ariel, 1990).

Beyond that, speakers can also realize visual anaphoric reference through speech-accompanying gestures (McNeill, 1992; Kendon, 2004). The current study asks whether addressees are sensitive to the use of speech-accompanying gestures when they are used to track referents in discourse, and specifically, when gestures use recurrent locations to refer back to referents.

Various studies have described localizing gestures as playing an important role in reference tracking (e.g., McNeill and Levy, 1993; Gullberg, 1998, 2003, 2006; Kendon and Versante, 2003; So et al., 2009). Speakers tend to assign a location in gesture space to a referent at its introduction by way of a localizing gesture. They can then reuse this location when the referent is referred back to later in discourse. The second gesture is what we call a localizing anaphoric gesture (see Figures 1a,b for an example of two congruent localizing gestures in spontaneous production. The example is taken from a corpus of speech-gesture production, collected by the first author for another study) (see Supplementary Materials File ‘Consent form 2’, which has been signed by all participants appearing in all Figures).

**FIGURE 1a:**
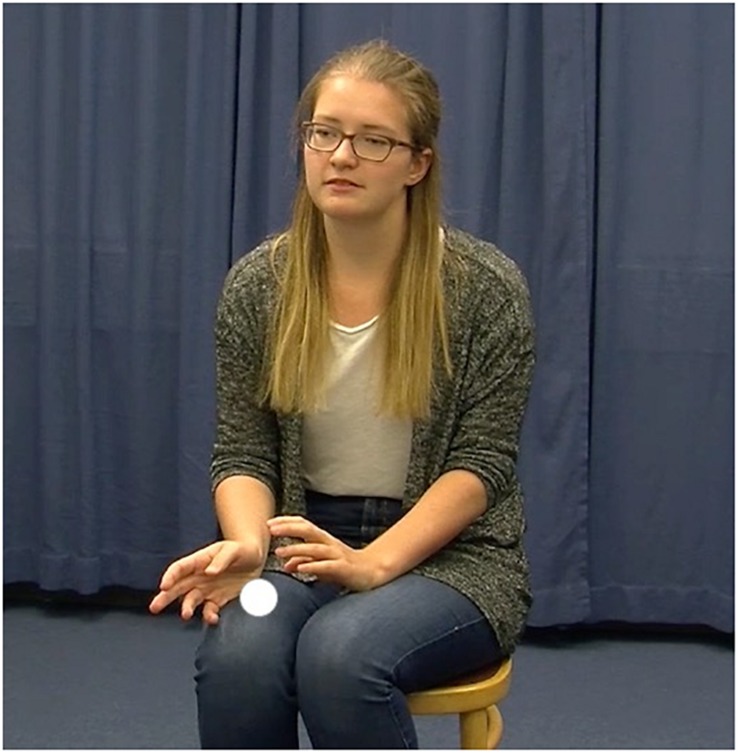
*Und **der erste Mann** nimmt ein’n schwern Stein* (“And **the first man** takes a heavy stone”). Example of a localizing gesture at a referent’s introduction (Words in bold are aligned with gesture stroke phase). This Figure shows a gesture which indicates a spatial area (indicated by the dot in white) above the speaker’s right knee for the referent at its introduction.

**FIGURE 1b:**
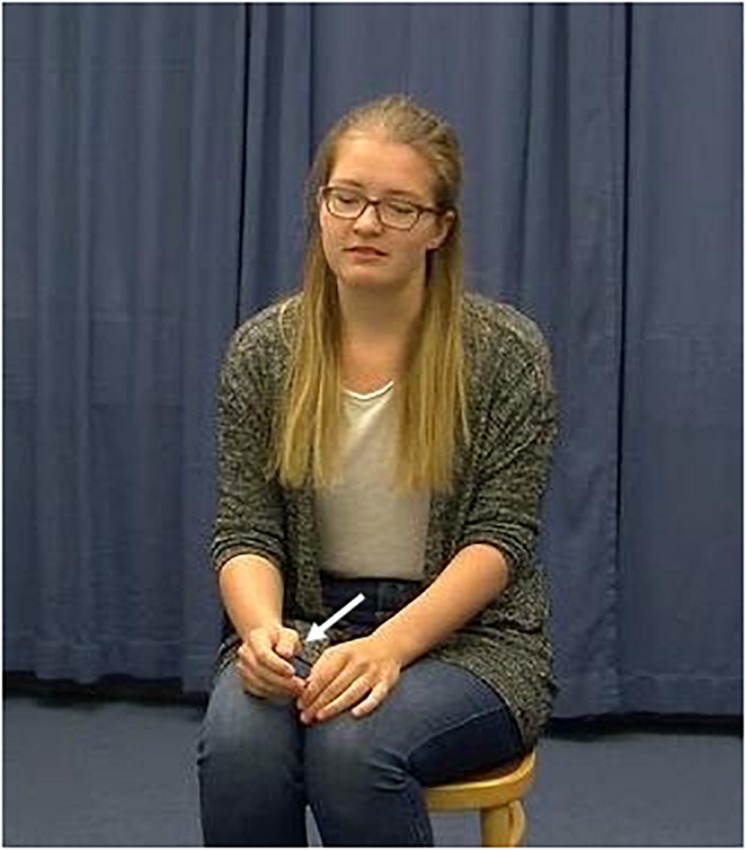
*Ähm der **Mann** hebt dann die Hand* (“Uhm the **man** then raises his hand”). Example of a localizing anaphoric gesture at a referent’s reintroduction after a gap of absence (Words in bold are aligned with gesture stroke phase). This Figure shows a gesture which places the referent exactly in the same area at its reintroduction after a gap of absence (the white arrow indicates the gestural movement toward the spatial area).

Importantly, the use of localizing anaphoric gestures depends on the discourse context. When speakers maintain a referent, they are less likely to align a gesture with the spoken referential expression (often a pronoun). But after a gap, when speakers need to reintroduce a referent (often using a richer nominal form), they frequently also accompany the mention of the referent with a localizing anaphoric gesture (Gullberg, 2006). Thus, gestures reflect the information status of a referent in parallel with speech. In production, less marking material is used for highly accessible referents (pronominal forms + absence of gesture), and more marking material is used for the reactivation of referents (nominal forms + localizing gestures) across both modalities (i.e., speech and gesture; Marslen-Wilson et al., 1982; Levy and McNeill, 1992; McNeill, 1992; McNeill and Levy, 1993; Gullberg, 1998, 2003, 2006; Yoshioka, 2008; Perniss and Özyürek, 2015; Debreslioska and Gullberg, 2019). Interestingly, some production studies also suggest that parts of the gestural reactivation process are meant for the listener. For instance, Gullberg (2006) showed that speakers adhered more consistently to locations set up by localizing gestures when addressees could see them than when they could not (i.e., when speakers and addressees were separated by a screen preventing eye contact and gesture visibility). Furthermore, Gullberg (1998) and Gullberg (2011) showed that in interactive stretches, some addressees even pointed back to locations previously established for referents by the speakers. This is interpreted as evidence that addressees understand when spatial representations of referents were created.

A wealth of perception studies on cross-modal information integration support the fact that addressees integrate information from gestures with the meaning in speech. Evidence for this view comes from behavioral studies (e.g., Graham and Argyle, 1975; Riseborough, 1981; Thompson and Massaro, 1986, 1994; Beattie and Shovelton, 1999; Kelly et al., 1999, 2010b), ERP studies (e.g., Kelly et al., 2007; Özyürek et al., 2007; Sheehan et al., 2007; Wu and Coulson, 2007); and fMRI studies (e.g., Skipper et al., 2007; Holle et al., 2008; Dick et al., 2014; see also Kendon, 1994; Hostetter, 2011 on the communicative function of speech-accompanying gestures).

Perception studies specifically testing the processing of localizing anaphoric gestures also generally support this view. Some studies suggest that localizing gestures that are spatially congruent with previous gestures can facilitate processing in comparison to spatially incongruent localizing gestures (Cassell et al., 1999), while others suggest that congruent localizing gestures facilitate processing in comparison to speech alone (Gunter et al., 2015; Gunter and Weinbrenner, 2017). Finally, anaphoric localizing gestures are shown to reinforce expectations about referent resolution in speech (Goodrich Smith and Hudson Kam, 2012; Nappa and Arnold, 2014), and to help identify referents (Sekine and Kita, 2015).

However, there are many inconsistencies within and across those studies, which also suggest addressee insensitivity. For instance, in Cassell et al. (1999) participants retold taped narratives in which they had seen a speaker either use localizing gestures congruently or incongruently. They produced more retelling inaccuracies in the incongruent than in the congruent condition. Interestingly, however, only 32% of all incongruencies resulted in retelling inaccuracies. Thus, although an effect was observed in comparison to the congruent condition, participants were also very likely not to be influenced by the incongruent information provided in gesture (68% of the time).

Similarly, Hudson Kam and Goodrich Smith (2011) found that addressees are insensitive to gesturally established locations for entities in narratives. They showed participants taped narratives in which a speaker used (multiple) congruent localizing gestures for each of two entities, placed left and right. Participants did not adopt a consistent perspective when asked to choose one of two pictorial representations of the story. The pictures were always mirror images of each other showing one entity on the right and the other on the left. In another study, Goodrich Smith and Hudson Kam (2012) used similar taped narratives, but in a critical clause, the speaker used an “ambiguous” pronoun (i.e., a pronoun that could refer to either of the two same gendered preceding referents) with a localizing gesture that either matched the first or second protagonist. They found that participants preferred the first protagonist as referent for the pronoun when no gestures were used, replicating the order-of-mention effect, a well-established cue for pronoun resolution in many spoken languages (e.g., Gernsbacher and Hargreaves, 1988). The presence of gestures indicating the second protagonist changed this pattern, and participants chose the second participant more often (38%). Importantly, however, they still chose the first mentioned protagonist even more often (44% of the time; and 18% of the time, participants did not choose either of the two relevant referents).

Reaction time experiments using comparable designs further show diverging results. On the one hand, Nappa and Arnold (2014) found that addressees profit from gestures that reinforce expectations coming from speech (as in order-of-mention for pronoun resolution), leading to faster responses. But they also showed that addressees were not influenced by gestures that went against expectations drawn from speech, that is, their performance was not slowed down by incongruently used gestures. In contrast, Sekine and Kita (2017) found the opposite. In comparison to speech alone, addressees were slowed down in the incongruent condition, but they were not faster to respond in the congruent gesture condition.

Gunter and Weinbrenner (2017), examined event-related brain responses in participants who watched videos of a person talking about topics of a dualistic nature, introducing, and referring back to each topic multiple times by gesturally placing them left and right in gesture space. The results suggested a difference in activation patterns when brain responses to critical expressions accompanied by congruent gestures were compared to those with no gesture, but showed no difference when congruent, incongruent and no gesture conditions were compared.

The contradictory results in these studies may be due to the underlying assumption about the function of anaphoric gestures and to certain methodological choices. Here, we discuss five important points. First, the natural alignment of speech and gesture in the context of reference tracking is not taken into account in every study. Production studies show that speakers tend to produce gestures in alignment with nominal forms in reintroduction contexts (e.g., Gullberg, 2006). In discourse, localizing anaphoric gestures thus typically do not have a disambiguating function when it comes to referent identification (e.g., Gullberg, 2006; So et al., 2009), and they typically do not occur with pronouns. In many experiments, however, this is how gestures are used in the stimulus materials with localizing gestures co-occurring with (ambiguous, as defined above) personal pronouns (e.g., Goodrich Smith and Hudson Kam, 2012; Nappa and Arnold, 2014; Sekine and Kita, 2017).

Second, there is an overemphasis on contrast. Most studies work with contrast between two referents located in two opposite locations (e.g., Goodrich Smith and Hudson Kam, 2012; Nappa and Arnold, 2014; Gunter and Weinbrenner, 2017; Sekine and Kita, 2017). This choice means that an incongruent localizing gesture for one referent is always produced in the space previously assigned to the other referent. The underlying assumption seems to be that localizing gestures that are incongruent with a referent locus should always be produced in a space that has already had a meaning assigned to it. It is therefore unclear how a gesture produced in an unassigned location may affect comprehension.

Third, there is a confound of handedness. In all studies, narrators use their right and left hands to locate referents to the right and left in gesture space, respectively. This experimental choice means that it is hard to disentangle which gestural level of representation is crucial for addressees’ processing difficulties or enhancement. That is, it is unclear whether it is handedness, location, or both that matter for reference tracking. Location and handedness are generally considered to be two different dimensions of gestural representation processes in discourse (e.g., McNeill and Levy, 1993). The underlying assumption about the difference between handedness and the use of space for reference tracking strategies can be explained as follows. If an addressee associates a hand with a referent, then the location of the hand might matter less (or not at all). That is, if “a hand is the referent,” then the addressee may always retrieve the representation of the referent when that hand is being used, regardless of in which part of space the gesture is produced (or the hand is located). However, if an addressee associates a location with a specific referent, then it may not matter which hand points back to that location. Previous studies have attributed their results to both dimensions. For instance, while Cassell et al. (1999) attribute the effect in their study to handedness, suggesting that addressees associate each hand with a different referent, other studies assume location in space to be the determining factor (e.g., Goodrich Smith and Hudson Kam, 2012; Gunter et al., 2015; Sekine and Kita, 2015, 2017; Gunter and Weinbrenner, 2017). However, none of the studies provide decisive evidence either way.

Fourth, there is potentially altered allocation of attention to gesture. Some studies have chosen to blur (Gunter et al., 2015; Gunter and Weinbrenner, 2017), or cover the narrators’ faces with masks (Sekine and Kita, 2015, 2017). While this technique might control important aspects of an experiment (e.g., being able to use the same audio for different videos), it also means that participants’ attention to the gestures may be increased. There is evidence that addressees typically focus their gaze on the speaker’s face and only process gesture input in peripheral vision (Gullberg and Holmqvist, 1999, 2006). However, with the face masked or blurred, attention allocation is likely to be altered toward gestures. In addition, in some of the same studies (Gunter et al., 2015; Sekine and Kita, 2015, 2017), the gestures were produced at shoulder height, which also draws more attention to them, considering that this is a rather marked area for gesture production (McNeill, 1992 for coding scheme of gesture space; Müller, 1998). In Sekine and Kita (2015, 2017) the narrators further used marked resting positions for the hands after they had performed the gestures. That is, when narrators had gesturally introduced referents by locating them, they held their hands in those spaces (at shoulder height) for the rest of the narrative. This might lead to over-specification since the locations were kept active throughout the narrative.

Finally, there is a lack of distractors and control of possible learning effects. Only two studies report using distractor items or items with gestures fulfilling other functions in relation to speech (Cassell et al., 1999; Goodrich Smith and Hudson Kam, 2012). By not including distractors, studies may have increased participants’ awareness of the topic being studied, that is, drawn attention to gestures with a referential function more generally, and possibly even to location/handedness of gesture in particular. This is especially important in experiments in which only a congruent condition was compared to a no gesture condition (Gunter and Weinbrenner, 2017, Experiment 2). Such a design might have led participants to learn over the course of the experiment that all gestures reliably have the same function because they always provided the same information, and thus that the gestures have to be useful for the task at hand.

In contrast to previous studies, the current experiments focus on a more naturalistic setting, in which the speaker’s face can be seen and gestures are produced in central gesture space (i.e., between chest and hip height and relatively close to the body on the left and right), while still controlling for handedness and learning effects. Moreover, the current study goes beyond previous research by testing participants’ sensitivity to anaphoric gestures in the context of a single gesturally tracked referent. The study therefore takes a first step toward addressing the potential methodological confounds discussed above.

## The Current Study

The present study examines whether addressees are sensitive to the use of localizing anaphoric gestures. We conducted two reaction time experiments with differing tasks comparing performance in three conditions: gesture congruent, gesture incongruent, and no gesture. The same stimulus narratives were used in both experiments. In the gesture congruent condition, a referent is introduced with a localizing gesture in utterance 1 and reintroduced after an intervening utterance by a localizing anaphoric gesture in utterance 3. In the incongruent condition, the referent is reintroduced by a localizing gesture in a different, previously unassigned location in space (note that, by our definition an incongruent gesture is not technically an anaphoric gesture; see also Gullberg, 2006). We also added a no gesture condition as a baseline condition in both experiments. The results from the comparisons between the gesture and no gesture conditions need to be considered very cautiously though, because they are not perfectly comparable. In the gesture conditions, the spoken referential expressions are aligned with the stroke phase (or the most meaningful part) of the localizing gestures. However, gestures have a preparatory phase that precedes the stroke, and therefore typically start before the referential expressions are uttered. Thus, gestures might provide information to addressees before the referential expressions are even produced. This is, of course, not the case in the no gesture condition.

In Experiment 1, the task for participants was to answer a question about an action performed by the tracked referent in a fourth and critical utterance. The assumption was that a preceding congruent localizing gesture should facilitate responses to the content question, whereas a preceding incongruent gesture could render decisions regarding the referent more difficult (cf. Sekine and Kita, 2017 for a similar task). In Experiment 2, the participants saw the referent to be tracked in written form before the start of the narratives. Their task was to press a key as fast as possible every time they encountered the referent during the subsequent narrative. The assumption was that congruent gestures speed up the recognition of a bimodal anaphoric expression, whereas incongruent gestures slow it down.

In addition, the present study differs theoretically and methodologically to the existing literature on bimodal anaphor perception in a number of ways. First, the narrator gesturally tracks only one referent rather than two. The assumption is that if addressees indeed associate a certain location with a discourse referent, then that will be the case even if there is no contrast between that referent and another. Second, the referent is located twice in the narrative, once at its introduction and once at its reintroduction, respecting the discourse context in which localizing anaphoric gestures are typically found in production. The assumption is that addressees can create a spatial representation of a referent in a minimal context, even after only two instances of localization (cf. Sekine and Kita, 2017). Third, the narrator always uses two hands to locate a referent rather than one in order to exclude handedness as a potential confound for referent assignment. Fourth, the narrator’s face is visible and the gestures are produced in central gesture space (cf. Gullberg and Kita, 2009). Finally, we added distractor items with gestures fulfilling other functions to obscure the goal of the study.

## Experiment 1

We test the hypothesis that addressees are sensitive to the use of spatial localizing anaphoric gestures. Following previous research, addressees may profit from the use of a recurrent location for a discourse referent when processing narrative discourse. Therefore, we predict (a) that participants are faster in the congruent condition than in the incongruent condition. In relation to the no gesture condition, we predict (b) that participants will be faster in the gesture congruent condition, and slower in the gesture incongruent condition.

### Materials and Methods

#### Participants

Twenty-eight^1^ students enrolled at DEKRA Hochschule, Berlin, Germany, participated in the study (mean age 23; 19 female). All participants were native speakers of German who had grown up monolingually. We recruited participants through notices at the school, and word of mouth. They received a small fee for their participation in the study.

### Stimuli/Materials/Design

The experimental stimuli were 50 video-taped narratives told by a female native speaker of German. She produced ten narratives without gestures, 20 with congruent gestures, and 20 with incongruent gestures. The 20 narratives in the congruent condition were the same as the 20 narratives in the incongruent condition. The speaker was trained to perform narratives and accompanying two-handed localizing gestures as naturally as possible. She was also trained to keep the rest of her body as still as possible, keep the intonation of her speech as similar as possible, and to speak at a comparable speed across all narratives. The speaker was recorded sitting in a chair with no armrests against a plain, dark blue background (see Figures 2a,b–3a,b). She performed all gestures in central gesture space (coded as “center right and left” in McNeill, 1992; cf. Gullberg and Kita, 2009) because this corresponds to the typical culture-specific area for German speakers (Müller, 1998).

**FIGURE 2a:**
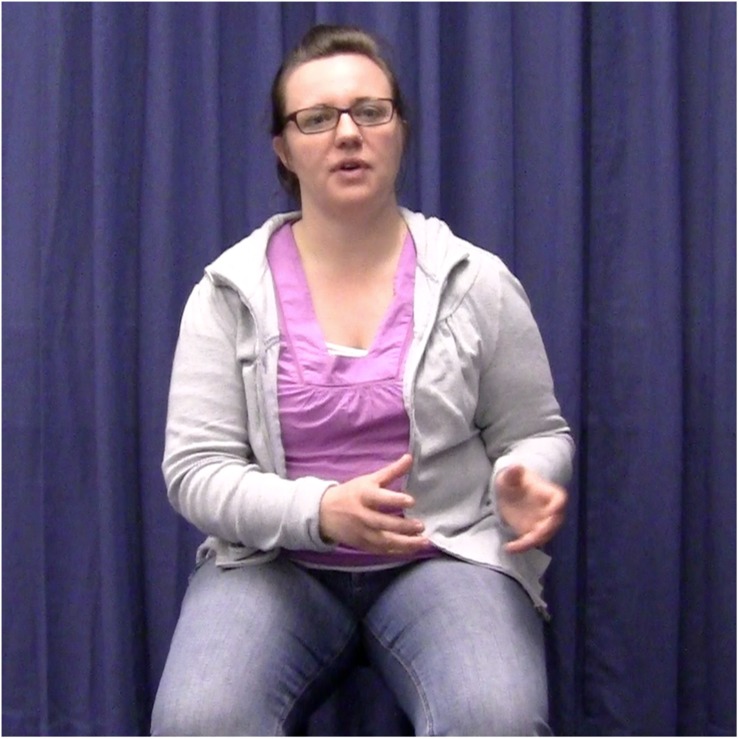
Example of the gesture congruent condition. The speaker introduces the referent in clause 1 by using a localizing gesture to the left.

**FIGURE 2b:**
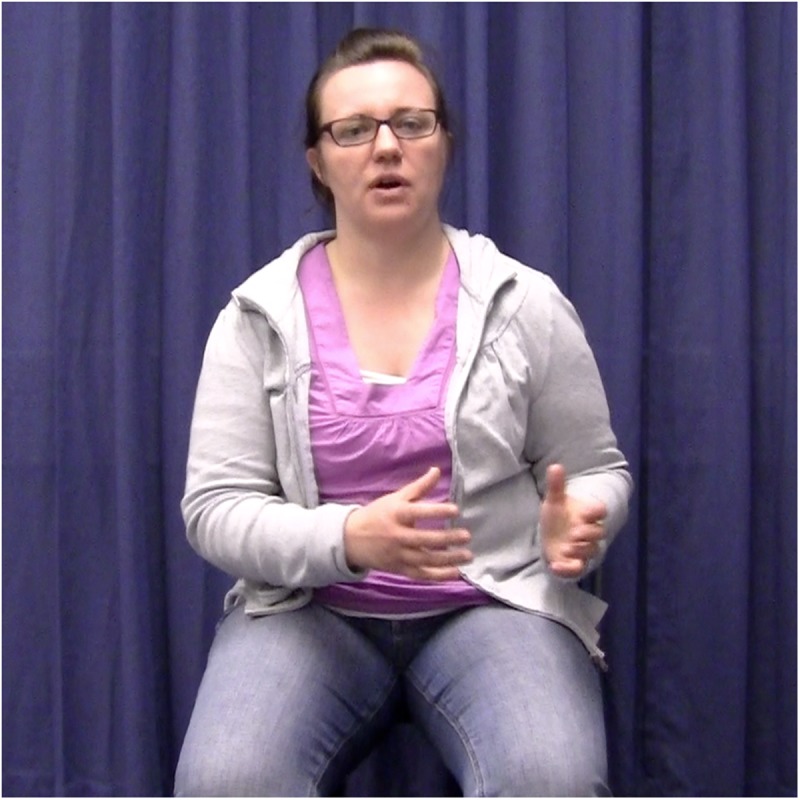
Example of the gesture congruent condition. The speaker reintroduces the referent in clause 3 by using a congruent localizing anaphoric gesture.

**FIGURE 3a:**
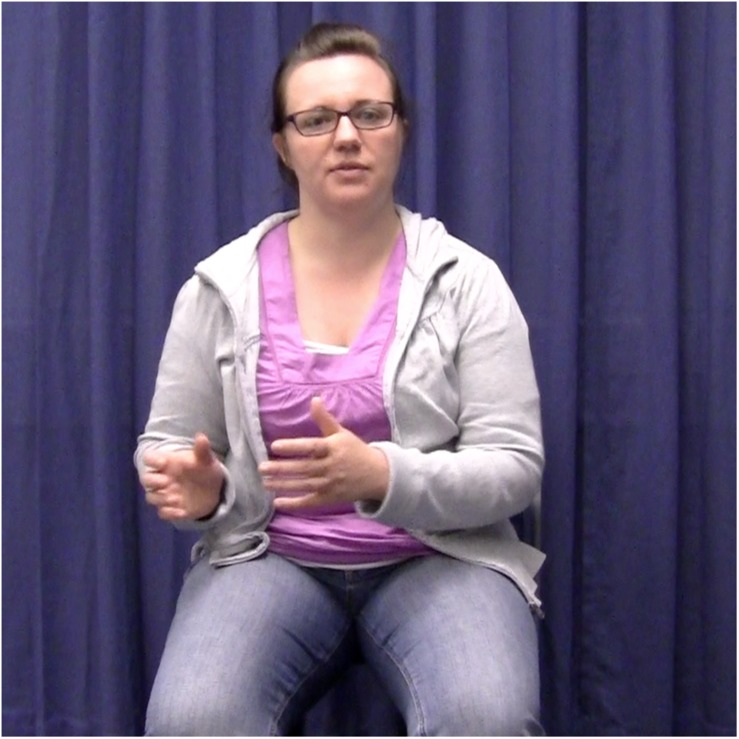
Example of the gesture incongruent condition. The speaker introduces the referent in clause 1 by using a localizing gesture to the right.

**FIGURE 3b:**
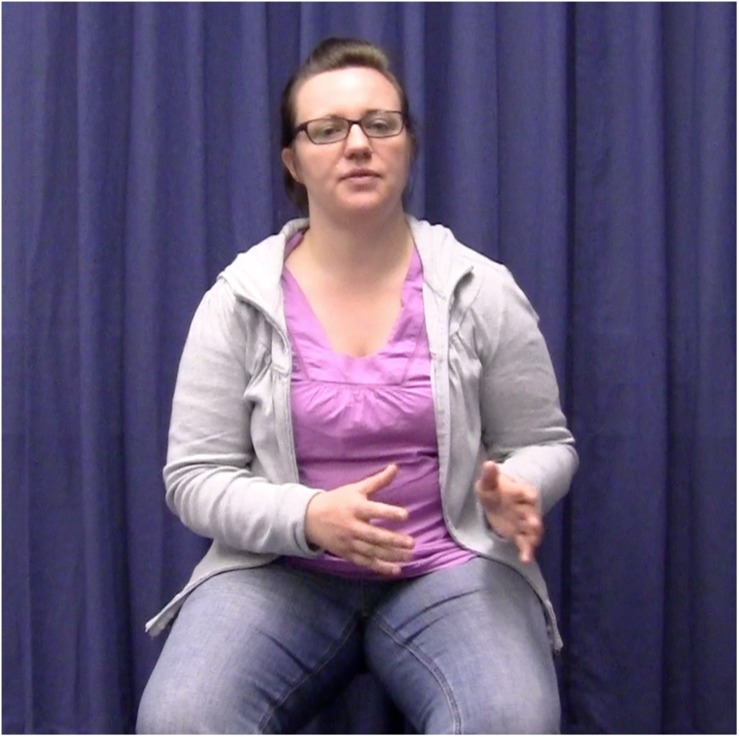
Example of the gesture incongruent condition. The speaker reintroduces the referent in clause 3 by using an incongruent localizing gesture.

All narratives consisted of 30–35 words, lasted between 8.7–11 s, and had the same utterance structure (example 1). In the first utterance, the main protagonist is introduced with an existential construction and an indefinite lexical NP as grammatical subject (e.g., “There was a woman”). The second utterance is about a secondary character (e.g., “husband”), who does not manage to carry out a certain task. In the third utterance, the main protagonist is reintroduced with a lexical NP as grammatical subject (e.g., “Then the woman…”), and it is explained how she intends to help the other character with the task. In the fourth utterance, the main protagonist either calls or writes to someone for assistance. This action corresponds to the relevant action verb that participants need to respond to (henceforth referred to as the “target verb”). The fifth and last utterance served as a wrap-up utterance. There were always 11 syllables between the anaphoric expression and the target verb (For a list of all stimulus narratives, see the Supplementary Materials File ‘Stimulus Narratives’). We measured the time (in ms) between the onset of the anaphoric expression and the onset of the target verb. The average time was 2,203 ms (*SD* = 176) in the congruent condition, 2,126 ms (*SD* = 136) in the incongruent condition, and 2,001 ms (*SD* = 37) in the no gesture condition. The time difference between onset of the anaphoric expression and the onset of the target verb was added as an additional predictor variable into our models for analysis in order to control for this variation (see analyses below).

In the experimental items, localizing gestures occurred in exact temporal alignment with the first and second referential expressions for the main protagonist. All gestures were performed with two hands, and specifically with the form illustrated in Figures 2a,b–3a,b. This gesture form is (frequently) used in spontaneous gesture production, and specifically also for locating referents in space (see Gullberg, 1998). In the gesture congruent condition, the first and second gestures were placed in the same location in space, half of the time to the right, the other half to the left. In the gesture incongruent condition, the second gestures were placed in the opposite locations in space, either left or right depending on where the first gesture was placed.

(1) *Da war* ***eine Frau^1^***. *Und ihr Mann konnte den Motor in seinem Auto nicht selbst reparieren. Also hat sich* ***die Frau^2^*** *dazu entschlossen, ihren Bruder anzu****rufen****/anzu****schreiben***. *Der soll ihm dann zur Hilfe kommen.*
  ‘There was a woman^1^. And her husband couldn’t repair the engine of his car by himself. So, the woman^2^ decided to call/write to her brother. He should come to help him out.’

^1^Gesture placed in right/left gesture space.
^2^Gesture placed in right/left gesture space.

**FIGURE 4:**
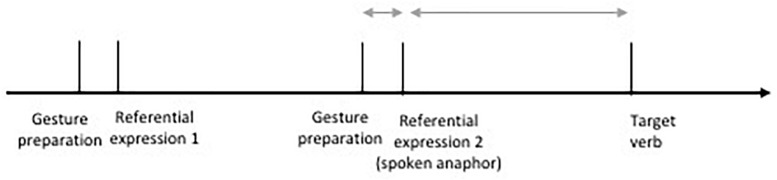
Time line of experimental items in gesture congruent and gesture incongruent conditions.

Gesture preparations started between 200 and 680 ms before the onset of the spoken anaphoric expression (see Figure 4). Gesture preparations started slightly earlier in the gesture incongruent (*M* = 542 ms, *SD* = 103 ms) than in the gesture congruent condition (*M* = 408 ms, *SD* = 118 ms). The time difference between onset of the gesture preparation in relation to the onset of the spoken anaphoric expression was added as an additional predictor variable into our models for analysis in order to control for this variation (see analyses below).

Referential expressions used for the main protagonists were the common nouns *Mädchen* “girl” and *Frau* “woman” for the gesture conditions, and *Junge* “boy” and *Mann* “man” for the no gesture condition. We also always added two other animate characters to the stories in order to avoid a contrastive context between two (animate) referents, which is what previous studies have typically used. Furthermore, our goal was to create stimuli that reflect reasonably natural discourse where it is not uncommon for people to speak about three animate referents when telling a story.

We also created 30 distractor narratives that differed from the experimental narratives in various ways. Half of the distractor items differed in the spoken clausal structure for the introduction and reintroduction of protagonists; the other half retained the structure of the experimental items. In half of the distractor items, the introductions of referents were accompanied by two-handed localizing gestures as in the experimental items, whereas in the other half the introductions of referents were not accompanied by gestures. In all distractor items the narrator also naturally performed other gestures, mostly depicting actions or simple beats, that were aligned with (and thus highlighted) parts of speech other than the relevant referential expressions. Distractor items also varied in length, and crucially, differed in terms of where the target verb was mentioned. This was done in order to ensure that participants stayed attentive to the content of the speech at all times.

We created two versions of the experiment each with 30 experimental items (10 gesture congruent, 10 gesture incongruent, 10 no gesture items) mixed with 30 filler items. Each participant saw an experimental narrative in only one version, congruent or incongruent. Between participants the versions were counter-balanced. In the experiment, experimental trials alternated with distractor trials. Otherwise, the order of the trials was randomized.

#### Post-processing and Stimulus Selection

We used a Canon Legria High Definition16E consumer camera to tape the narratives. The recording format was AVCHD. The videos were transformed into .mpg files with a frame rate of 25 frames per second and a resolution of 1920 × 1080 and edited in Adobe Premier Pro video editing software (cropping, cutting beginning and end of videos, color adjustment for normalization purposes).

Each narrative was videotaped 10–15 times to allow the actor to practice and perform as naturally as possible. One criterion for selecting the best instance of each item was that spoken referential expressions overlapped in time with the localizing gestures. That is, those videos in which a gesture did not exactly align with a referential expression were excluded/not considered as stimuli. In the stimulus items, the alignment between gesture and referential expression was always *exact*. For instance, a gesture would align with the referential expression “a woman” and “the woman” in “There was a woman. […] So, the woman decided to call/write to her brother.” However, it is important to note that the gesture did not always span over the same syllables within the same referential expression. For instance, in some cases, the gesture stroke might have aligned with “the wo” and in others with “woman.” This natural variation on the stimulus material is to be expected and still constitutes exact alignment at the referential expression level. It is also accounted for in the statistical models (see section Analyses). Another criterion for selection of the best instance of each experimental item was that gesture handshape and location in space should correspond between the first and second gestures (Figures 2a,b–3a,b).

We analyzed the recordings in the video annotation software ELAN (Sloetjes and Wittenburg, 2008) and identified the gesture stroke, defined as the expressive and meaningful part of the gesture movement, to determine whether or not the stroke phase temporally aligned with the corresponding spoken referential expression. The narrative was excluded if this was not the case. At least one syllable of a relevant referential expression had to be temporally aligned with the time it took the speaker to perform the stroke phase (cf. McNeill, 1992). Other parameters, such as intonation, blinking, head position of the speaker or movement of other body parts were carefully observed, and those narratives that matched each other as closely as possible on all parameters were selected as stimuli.

#### Procedure

The experiment was carried out in a quiet room at the university. The clips were presented on a laptop running E-Prime version 2. The room was darkened (blinds down at all times) in order to avoid differences in lighting during the day and possible reflections on the screen. The experimenter first orally introduced the experiment. Participants then read specific instructions on paper. Their task was to watch the videos of the narratives carefully and, for each narrative, respond to the question “Did the main protagonist call someone for help?” as fast and accurately as possible by pressing the keys *j* for “yes” (*ja*) or *f* for “no” (*falsch*) on the keyboard. No explicit mention was made of the gesture information. The task implicitly probed the processing of information related to the referent. This task was chosen to avoid conscious and strategic processing of the gesture and its referent in speech (cf. Kelly et al., 2010a, b). Participants were specifically encouraged to press the button as soon as they knew the answer and not to wait until the end of the video.

The correct answer was yes for half of the narratives and no for the other half (ending with *write* instead of *call*, see example 1). The instructions included an explanation that the main protagonist was always the first mentioned character, and that the narratives were about a problem that this protagonist had to solve. The instructions further contained three examples of narratives with corresponding correct responses and explanations, mirroring the difference between experimental items and two kinds of distractor items.

The experiment lasted 10–15 min, after which participants filled out a consent form (see Supplementary Materials File ‘Consent form 1’), and a language and background information questionnaire. The experimenter debriefed participants verbally.

We had to exclude data from 4 participants because more than a third of their responses were incorrect or given after the narratives had ended. The analyses were performed on the remaining 24 participants.

#### Analyses

We fitted linear mixed effects models with the lmerTest package (Kuznetsova et al., 2017) in RStudio (RStudio Team, 2016) to the participants’ response times. Response times were time locked to the onset of the relevant part of the target verb (*rufen* “call” or *schreiben* “write”; see example 1). We excluded 16 incorrect trials (4 congruent, 6 incongruent, 6 no gesture) from the analysis (i.e., when participants responded incorrectly to the question). Furthermore, we excluded three responses that were given earlier than 100 ms after the onset of the target verb, and 24 responses given after the narrative had ended, corresponding to a total of 5.9% of the data.

The predictor variables were (1) experimental condition (congruent, incongruent, no gesture), (2) the time difference between onset of gesture preparation and onset of spoken anaphoric expression, (3) the time lag between onset of spoken anaphoric expression and onset of target verb, and (4) trial number. We also added random intercepts for each subject. Note that we also ran models with random intercepts for each experimental item, but since there was no difference between the models, we report only on the simpler ones here (information about the additional models is provided in Appendix A).

It is important to control for (2) the difference between the onset of the gesture preparation in relation to the onset of the spoken anaphoric expression since it is well-known that gestures usually start before the onset of the expression to which they are semantically related (e.g., Kendon, 1972; Schegloff, 1984). Thus, an anaphoric gesture might provide information about which entity will be mentioned before the spoken expression itself has been produced and before the gesture stroke has begun. Moreover, since there is natural variation between the different items in our material, it is important to take that into account. There is also natural variation in terms of (3) the temporal distance between the anaphoric referential expression and the target verb which also needs to be controlled for.

Since (2) the time difference between onset of gesture preparation and onset of spoken anaphoric expression, only applies in the two gesture conditions, we ran two analyses. In the first analysis, we compare the two gesture conditions (congruent versus incongruent) including (2). In the second analysis, we compare the two gesture conditions to the no gesture condition by excluding variable (2) the time difference between onset of gesture preparation and onset of spoken anaphoric expression. In this analysis, the no gesture condition is coded as the intercept in the model. We report the estimates derived from the analyses in the tables.

### Results

#### Comparison Between the Gesture Congruent and Gesture Incongruent Conditions

First, we examined the response times in the two gesture conditions. Table 1 shows the estimated response times per condition derived from the analysis. The results suggest that participants were faster to respond in the gesture incongruent than in the gesture congruent condition (*EST* = −106.4, *SE* = 37.37, *t*= −2.85, *p* = 0.005). There was no significant effect of (2) the time difference between onset of gesture preparation and spoken anaphoric expression (*EST* = −0.04, *SE* = 0.16, *t*= −0.27, *p* = 0.789), suggesting that it did not matter when the gesture preparation started in relation to the spoken anaphoric expression for the response times. There was also no significant effect of (3) the time lag between onset of anaphoric referential expression and onset of target verb (*EST* = −0.01, *SE* = 0.11, *t* = 0.07, *p* = 0.949), suggesting that the variation in distance between the anaphoric expression and target verb also had no influence on participants’ response times. There was a marginally significant trial effect (*EST* = −3.65, *SE* = 1.89, *t* = −1.93, *p* = 0.054).

**TABLE 1:** Response time estimates with 95% confidence intervals derived from models in Experiment 1.

	**Congruent**	**Incongruent**	**No**
**Condition**	**gesture**	**gesture**	**gesture**
Analysis 1: RT in ms (95% CI limits)	861 (313–1409)	755 (201–1308)	–
Analysis 2: RT in ms (95% CI limits)	804 (346–1263)	693 (254–1133)	742 (326–1158)

#### Comparison Between the Gesture (In)Congruent and No Gesture Conditions

Next, we compared the gesture conditions to the no gesture condition. Table 1 shows the estimated response times per condition derived from the analysis. The results suggest that there was no difference between the gesture congruent and the no gesture condition (*EST* = 62.34, *SE* = 37.64, *t* = 1.66, *p* = 0.098). There was also no difference between the gesture incongruent condition and the no gesture condition (*EST* = −48.65, *SE* = 33.45, *t* = −1.45, *p* = 0.146). Further, there was no effect of (3) the time lag between onset of anaphoric referential expression and onset of target verb (*EST* = 0.02, *SE* = 0.10, *t* = 0.20, *p* = 0.844), again suggesting that this variation had no influence on participants’ response times. Finally, there was an effect of trial number (*EST* = −4.86, *SE* = 1.50, *t* = −3.25, *p* = 0.001), suggesting that participants responded 4.86 s faster at their last trial than at their first.

### Discussion

The results from Experiment 1 show that, contrary to predictions, participants were faster to respond in the gesture incongruent than in the gesture congruent condition. There were no significant differences between any of the two gesture conditions and the no gesture condition. These results seem to suggest that incongruent localizing gestures might facilitate processing speed in comparison to congruently used (i.e., anaphoric) localizing gestures. No previous studies have reported an advantage of the incongruent condition in comparison to the congruent condition. We can therefore only speculate as to the reasons for this result. A first possibility is that incongruent gestures help addressees because they are more marked and noticeable than congruent gestures, which in turn draw less attention and are less noticeable. This interpretation is related to a second but linked option, namely that addressees may have a high level of acceptance for incongruent locations because speakers use them frequently in spontaneous face-to-face interaction (see further in General Discussion). Finally, a third possibility is that congruent localizing gestures caused a processing cost, perhaps because they were perceived as overexplicit. Since the two congruent localizing gestures were used in a rather short piece of discourse with minimal requirements for referent reintroduction in speech (i.e., one intervening utterance containing one intervening new referent as grammatical subject), it is possible that participants did not expect an anaphoric gesture and therefore perceived them as overly explicit (see further in section General Discussion).

Finally, the unexpected results may be due to the fact that Experiment 1 failed to directly measure the processing of referential expression + localizing gesture. Since there was a relatively long temporal distance between the (in)congruent localizing gestures and the target verbs in the stimulus narratives, it is possible that the effect of the (in)congruent gesture had subsided by the time participants came across the target verb. This could also explain why there was no difference between the gesture and no gesture conditions. To probe this possibility, we conducted a second experiment with the same set of stimuli but with a different task in order to test the processing of referential expression + localizing gesture more directly by narrowing down the time span. Participants saw a referential expression in written form on the screen before a narrative started and were instructed to track the given referent by pressing a key each time they encountered it during the narrative. This task allows us to measure processing of spoken anaphoric expression ± (in)congruent gestures more directly, by examining how quickly participants recognize a (bimodal) anaphoric expression.

## Experiment 2

For Experiment 2, we test the same hypothesis as in Experiment 1, namely that participants profit from the recurrent use of a location for a gesturally tracked referent. We make the same predictions as in Experiment 1, namely that (a) participants perform faster in the congruent than in the incongruent condition; and (b) in comparison to the no gesture condition, participants perform faster in the gesture congruent condition, and more slowly in the incongruent condition.

### Materials and Methods

#### Participants

Twenty-nine native German speaking students enrolled as exchange students at Lund University, Lund, Sweden participated in the study (mean age 24; 21 female). All participants were native speakers of German who had grown up monolingually in Germany. All of them were international exchange students. They were recruited through social media groups for international students at the university, and by word of mouth. Participants received a voucher for their participation in the study.

#### Procedure

We used the same stimuli as in Experiment 1. Participants carried out the experiment on a stationary computer in E-prime software (version 3) at Lund University Humanities Lab. Before each clip, participants saw the target referent (e.g., girl, woman) written on the screen, indicating that this was the referent they had to track in the subsequent narrative. The instruction was to press the key *j* for “yes” (*ja*) as fast as possible once they encountered the referent. We intentionally avoided using the word “hear” in the instruction. For a third of the trials, a yes/no comprehension question appeared after the video clip. This question always related to details in the narratives. Participants responded to the questions by pressing the keys *j* for “yes” (*ja*) or *f* for “no” (*falsch*) on the computer keyboard. We added the comprehension questions to ensure that participants stayed focused on the content of the narratives. The experiment lasted approximately 15 min. After the experimental session, participants filled out a consent form and a language and background information questionnaire. The experimenter debriefed participants verbally.

We excluded data from two participants. One participant had answered more than a third of the comprehension questions incorrectly, the other one provided only 1 out of 10 responses in the no gesture condition. The analyses were performed on the remaining 27 participants.

#### Analyses

As in Experiment 1, we fitted linear mixed effects models with lmerTest package (Kuznetsova et al., 2017) to participants’ response times. We time locked response times to the onset of the spoken anaphoric expression. If participants provided a keypress after they had encountered an anaphor, we assumed that they had recognized the anaphor, and thus used that data point in our analysis. We excluded 14 responses from the no gesture condition because they were given before, or within 100 ms after, the onset of the spoken anaphoric expression (corresponding to 1.7% of the data). Participants further failed to detect (i.e., did not press a key) the anaphoric expression 44 times in total (7 in congruent, 14 in incongruent and 23 in no gesture), which corresponds to another 5.4% of the total data set.

As predictor variables we used (1) experimental condition (congruent, incongruent, no gesture), (2) the time difference between onset of gesture preparation and onset of spoken anaphoric expression, and (3) trial number. We also added random intercepts for each subject. As in Experiment 1, we ran two analyses. The first one compared the gesture conditions in order to include (2) the time difference between onset of gesture preparation and onset of spoken anaphoric expression, and the second analysis compared the gesture conditions to the no gesture condition excluding (2). Again, we ran the models with random intercepts for each experimental item, but since there was no difference between the models, we report only on the simpler ones here (but see Appendix B for information about the additional models).

### Results

First, we analyzed response times for anaphor recognition in the two gesture conditions. Table 2 shows the estimated response times derived from the analysis. The analysis revealed no difference between the gesture conditions, meaning that participants were equally fast at recognizing anaphoric expression + gesture in both the gesture congruent and gesture incongruent conditions (*EST* = −3.36, *SE* = 26.84, *t* = −0.13, *p* = 0.901). Furthermore, there was a significant effect of (2) time difference between onset of gesture preparation and onset of spoken anaphoric expression (*EST* = −0.22, *SE* = 0.11, *t* = −2.03, *p* = 0.043), suggesting that gesture preparations that started earlier than others in relation to the anaphoric expression did provided an advantage for anaphor recognition. There was also an effect of trial number (*EST* = −4.42, *SE* = 1.32, *t* = −3.35, *p* = 0.001), suggesting that participants’ response time decreased over the course of the experiment.

**TABLE 2:** Response time estimates with 95% confidence intervals derived from models in Experiment 2.

	**Gesture**	**Gesture**	**No**
**Condition**	**congruent**	**incongruent**	**gesture**
Analysis 1: RT in ms (95% CI limits)	651 (548–754)	647 (518–776)	–
Analysis 2: RT in ms (95% CI limits)	563 (511–614)	530 (471–583)	582 (530–633)

Finally, we compared response times in the gesture conditions to the no gesture condition (see Table 2 for estimated response times derived from the analysis). The results showed that participants were significantly faster to respond in the gesture incongruent condition than in the no gesture condition (*EST* = −51.51, *SE* = 23.48, *t* = −2.19, *p* = 0.029), but there was no significant difference between the gesture congruent and no gesture conditions (*EST* = −18.92, *SE* = 23.26, *t* = −0.81, *p* = 0.416). As in the previous analysis, there was also an effect of trial number (*EST* = −4.39, *SE* = 1.09, *t* = −4.04, *p* = 0.000), suggesting that participants responded significantly faster at their last trial than at their first.

### Discussion

The analyses in Experiment 2 revealed that gesture congruency did not affect recognition speed of anaphoric expressions; participants were equally fast to recognize anaphoric referential expressions accompanied by congruent or incongruent gestures. Importantly, predictor (2), that is the time difference between onset of gesture preparation and onset of anaphoric expression, indicated that the earlier the preparation phase of the gesture started, the faster participants responded, however, any possible location information provided by the gesture before the spoken anaphoric expression started did not matter.

In contrast to the no gesture condition, we found that participants performed significantly faster in the incongruent condition than in the no gesture condition, but there was no difference between the no gesture and the gesture congruent condition. This result suggests that the presence of a spatially incongruent gesture matters more than congruence (i.e., when a recurrent location is used). Previous reaction time studies have either reported no difference between the incongruent and no gesture condition (Nappa and Arnold, 2014) or slower reaction times in the incongruent condition (Sekine and Kita, 2017). These studies, however, have worked with disambiguation and contrast, respectively, whereas in the present study only one referent was gesturally tracked. The incongruent location was previously unassigned and therefore arguably had no meaning. It is therefore difficult to directly compare the results of all three studies. Rather, the current experiment adds to the understanding of the phenomenon by showing that, in the context of gesturally tracking one referent, addressees’ processing seems to be enhanced by the presence of a gesture regardless of its spatial congruence in relation to a previous one. In fact, their spatial incongruence might even enhance addressees’ discourse processing.

## General Discussion

The aim of the current study was to examine whether addressees are sensitive to and/or profit from the use of localizing anaphoric gestures (i.e., the congruent condition) when processing a stretch of connected discourse. The results suggest that addressees are indeed sensitive to the use of localizing gestures, but in unexpected ways. Both experiments showed a lack of processing benefit of congruent gestures over incongruent gestures or a no gesture baseline condition. Instead, the results show that the incongruent condition speeds up performance in comparison to the congruent condition (Experiment 1), and to the no gesture condition (Experiment 2). The results from both experiments suggest similar interpretations, namely that when a single referent is tracked in the absence of ambiguity and contrast, spatially incongruent (anaphoric) gestures matter more than spatially congruent ones.

This initially surprising interpretation is supported by patterns found in spontaneous speech-gesture production. The natural input for addressees in face-to-face interactions appears to be rather imprecise and/or incongruent when it comes to localizing gestures. Production studies have convincingly shown that speakers reuse a congruent location for a referent previously assigned to a location in space less than half of the time (35% in So et al., 2009, and 42% in Gullberg, 2006). Thus, it is possible that addressees have a high level of acceptance for imprecise and/or incongruently used locations. In fact, spatially incongruent gestures may even have been more marked and noticeable, leading to facilitated discourse processing. We therefore assume that the incongruence manipulation in the current study was not perceived as such by addressees. We show a qualitative example from a data set of elicited narrative production to illustrate this point. Figures 5a,b show an example of an incongruent localizing gesture in spontaneous narrative speech-gesture production. In Figure 5a the speaker produces a gesture which indicates a spatial area to the speaker’s left for the referent “bridge” at its introduction (i.e., first mention). In Figure 5b the speaker reintroduces the referent “bridge” after a gap of multiple utterances and uses a gesture which places the referent to the right side of the speaker.

**FIGURE 5a:**
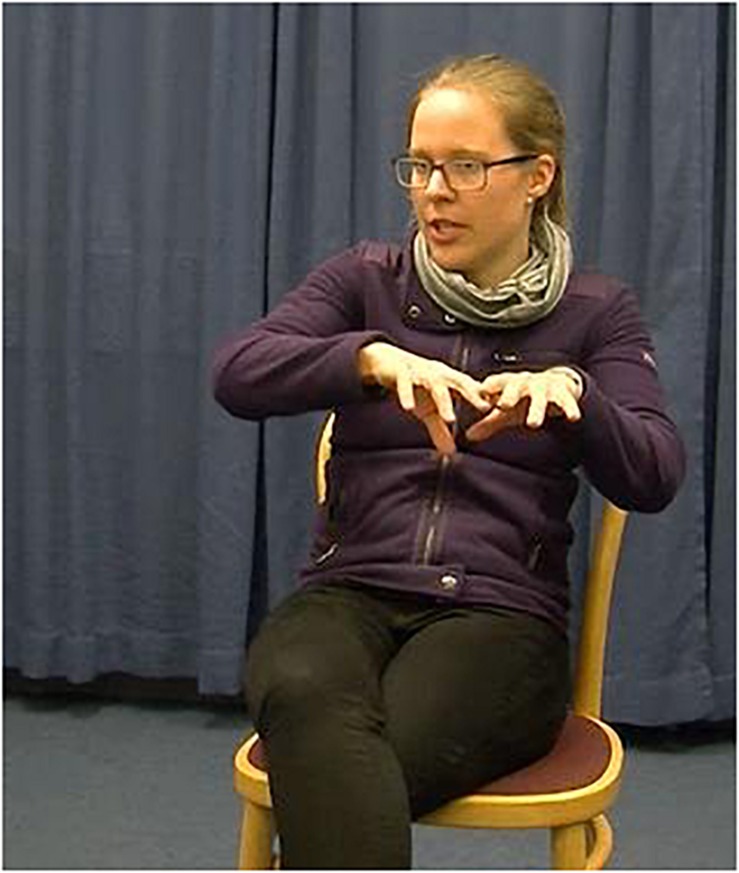
*Dann läuft er **auf diesen Steg** zu* (“Then he goes toward **this bridge”**). Example of a localizing gesture at a referent’s introduction (Words in bold are aligned with gesture stroke phase). The speaker mentions the referent “bridge” for the first time in this Figure.

**FIGURE 5b:**
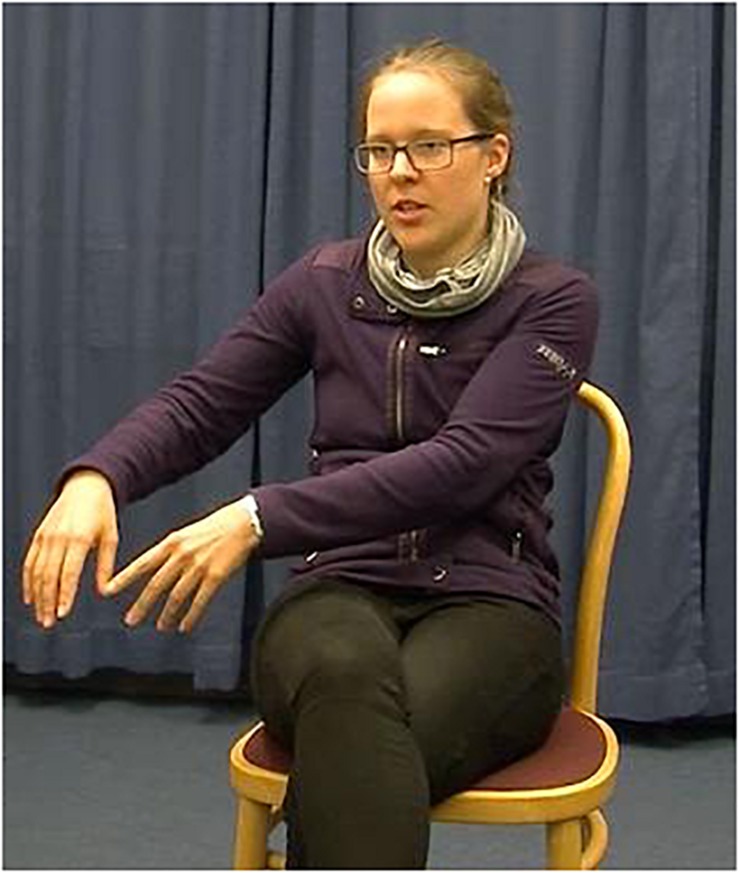
*Legt davor seine Sachen **aufn St***eg (“Puts his things **onto the bridge”**). Example of an incongruent localizing gesture at a referent’s reintroduction after a gap of absence (Words in bold are aligned with gesture stroke phase). The referent ‘bridge’ is mentioned again after a gap of absence of 3 clauses, but without an accompanying gesture, then there is another gap of absence of 8 clauses before the speaker mentions the referent ‘bridge’ again with a gesture indicating a spatial area to the speaker’s right.

A further interpretation of the current results is that gestures were perceived as overexplicit in the congruent condition. This would also explain why participants needed more or at least just as much processing capacity to integrate congruent gestures with spoken anaphoric expressions as incongruent gestures. Overexplicitness in speech refers to re-mentions of referents by the use of a noun when a pronoun would have sufficed. In speech perception studies, the repeated noun penalty effect (Gordon et al., 1993) predicts increased processing times for such overexplicit information (see also Vonk et al., 1992). We suggest that the use of localizing gestures in the congruent condition in the present study may also have been overexplicit. In fact, the two congruent gestures were used in the context of a rather short piece of discourse with minimal requirements for referent reintroduction in speech (i.e., one intervening utterance containing one intervening new referent as grammatical subject). However, the minimal context justifying a lexical noun phrase to reintroduce a referent in speech may not also be the minimal context for the use of an anaphoric gesture. Some qualitative studies on gesture production in discourse show that (localizing) anaphoric gestures are not only sensitive to the local information status of a referent, but also to bigger units, such as episode boundaries (Marslen-Wilson et al., 1982; Levy and McNeill, 1992; McNeill and Levy, 1993). Those studies indicate that an episode boundary might even be a stronger predictor for the occurrence of an anaphoric gesture. They suggest that more anaphoric gestures are used at the beginning of an episode (or at an episode boundary) than within episodes. Since in our stimulus material anaphoric gestures were used within an episode (with only one intervening utterance), it is possible that participants did not expect a congruent/anaphoric gesture to co-occur with the anaphoric expression and thus, perceived them as overexplicit. The longer processing times in the congruent condition could then reflect a *repeated gesture penalty*. Further gesture perception research is required to examine the effects of overexplicit gestures on comprehension to support a *repeated gesture penalty hypothesis*.

Finally, it may be possible that addressees did not interpret the second gestures in the experiments anaphorically as referring back to referents, but rather as referring to the new event (e.g., “the woman calling her brother”), since the second gesture occurs close to a discourse marker signaling a shift (“so”). Alternatively, addressees may also have interpreted the second gesture as referring to the new referent introduced in the third clause (e.g., “her brother”). This could be the case if addressees did not closely track the onset of a gesture (i.e., the second gesture could potentially refer to the new referent “brother” if the time lag between the mentioning of the two referents, “the woman” and “her brother,” is rather short; three words in the current material). Both these options are, in principle, conceivable for gestures with no iconic relationship to the referents with which they align, and/or for second gestures that use the opposite location (as in the incongruent condition). These explanations could potentially also explain the results in Gunter and Weinbrenner (2017). Future studies should test these possibilities by varying the alignment of gestures with referential expressions versus verbs versus other parts of the utterance.

The discrepancy between the results of the current study and previous research on this topic is mainly due to difference in design. In the present study, we used stimulus stories in which we matched production processes very closely, and we used only one referent that was gesturally localized and tracked in space. There was also no contrast or disambiguation worked into the narratives. Thus, our design is different from all previous studies on this topic. Therefore, we conclude that in a context, in which there is a contrast or in which a mismatch needs to be resolved, we can expect the congruent condition to enhance, and the incongruent condition to rather slow down processing (but see Gunter and Weinbrenner, 2017, Experiment 1). However, in a context in which location information is used as a means to map discourse onto space without any added disambiguating or contrasting function, the same expectation does not apply. Rather, the presence of a gesture seems to matter more than its spatial congruence, at least at its second appearance. In fact, spatially incongruent gestures might even be more noticeable in a context of one gesturally tracked referent (specifically because space has no differentiation function) and therefore help addressees more than spatially congruent gestures.

To further test this assumption, we must directly compare the gestural tracking of referents in an ambiguous/contrastive context versus a non-ambiguous/non-contrastive context for different numbers of referents. Furthermore, it is important to note that gesture research in the context of reference tracking with localizing gestures has not directly tested the contrast between spatial or non-spatial contexts. It therefore remains an empirical question whether participants would benefit more from gestural information when space is used in an abstract fashion versus when it is used topographically [i.e., when locations in gesture space are used as counterparts to physical locations in the (imagined) world]. In fact, Emmorey et al. (1995) found that American Sign Language users treat topographic locations differently from what in Sign Languages is called syntactic locations. Syntactic locations can be compared to the abstract use of space as we have designed it in the current study and as it has been used in previous research. It is possible that the function of localizing gestures differs in the two contexts. This can and should be pursued in future research. Finally, evidence about how precise gestural location information actually is in production is rather sparse (but see Gullberg, 2006). Further research should explore how consistent speakers typically are when tracking referents in different contexts. This type of enquiry would greatly deepen our understanding of the phenomenon and bridge the gap between production and perception studies.

## Conclusion

The results from the current study suggest that, in a context of a single gesturally tracked discourse referent, the presence of an incongruent (anaphoric) gesture is more useful to addressees than when a second gesture for the same referent uses a recurrent location in space. This interpretation is supported by speech-gesture patterns found in spontaneous production, which show that approximate/incongruent locations are rather common when it comes to gesturally tracking a referent. We also suggest that the relatively slow processing of congruent localizing gestures in the current and previous studies on this topic may be due to an over-explicitness of such repeated gestures in the tested contexts (*the repeated gesture penalty hypothesis*). This proposal will need further supporting research. Most importantly, the study highlights the importance of the context in which localizing anaphoric gestures are examined. The current results stand in contrast to previous studies that have mainly examined contexts in which anaphoric gestures fulfill a disambiguating or contrastive function. We conclude that gestures can be used to make discourse more coherent for addressees by paralleling reference tracking in speech but that the way gestures are deployed and integrated differs by context and number of referents.

## Ethics Statement

This study was carried out in accordance with the recommendations of the Swedish Research Council CODEX Rules and Guidelines for Research with written informed consent from all subjects. All subjects gave written informed consent in accordance with the Declaration of Helsinki. As per local legislation, an approval for this study was not required. The local ethics committee does not consider this type of study to encompass sensitive personal data and it was therefore exempt from an ethics review.

## Author Contributions

SD wrote the first draft of the manuscript. All authors were engaged in the editing and revision process, approved the publication of the content, and agreed to be accountable for all aspects of the work.

## Conflict of Interest Statement

The authors declare that the research was conducted in the absence of any commercial or financial relationships that could be construed as a potential conflict of interest.

## Footnotes

1 Sample sizes in both experiments were partly influenced by time and accessibility. Data collection for Experiment 1 was conducted in the field with a limitation in time, while data collection for Experiment 2 was conducted in Sweden with a limitation of accessibility to native German speakers with no/little knowledge of Swedish. In both experiments, the aim was to include at least 24 participants in the final analyses.
