# Supplementary material for: Addressees Are Sensitive to the Presence of Gesture When Tracking a Single Referent in Discourse
Source: Front Psychol. 2019 Aug 13;10:1775. doi: 10.3389/fpsyg.2019.01775 (PMC6700288; doi:10.3389/fpsyg.2019.01775)
Supplement: Supplementary file 1 [file Table_1.DOCX]

Appendices

**A**

1. Experiment 1, Summary of mixed effects model 1 (comparison of gesture congruent and gesture incongruent conditions)

*rt.lmer = lmer(RT~condition+Onset_RE2_Onset_target_word+Onset_prep_onset_RE2+*

*trial+(1|Subject),data)*

Random Effects

| **Group** | **Variance** | ***SD*** |
| --- | --- | --- |
| Subjects | 27676 | 166.4 |

Fixed Effects

| **Variable** | **Coeff. Estimate** | ***SE*** | ***t*** | ***p*** |
| --- | --- | --- | --- | --- |
| RE2 – target word | -0.00707 | 0.10960 | -0.065 | 0.94860 |
| G prep – RE2 | -0.04196 | 0.15658 | -0.268 | 0.78886 |
| Trial | -3.64696 | 1.89098 | -1.929 | 0.05446 |
| **Condition:** |  |  |  |  |
| Gesture congruent vs. Gesture incongruent | -106.40180 | 37.37686 | -2.847 | 0.00464 |

1. Experiment 1, Additional analysis 1 (comparison between gesture congruent vs. gesture incongruent conditions, including ‘item as random factor’)

*rt.lmer = lmer(RT~condition+Onset_RE2_Onset_target_word+Onset_prep_onset_RE2+*

*trial+(1|Subject)+(1|Item),data)*

Random Effects

| **Group** | **Variance** | ***SD*** |
| --- | --- | --- |
| Subjects | 28000 | 167.33 |
| Item | 9775 | 98.87 |

Fixed Effects

| **Variable** | **Coeff. Estimate** | ***SE*** | ***t*** | ***p*** |
| --- | --- | --- | --- | --- |
| RE2 – target word | - 0.007388 | 0.1524 | -0.048 | 0.9616 |
| G prep – RE2 | - 0.04059 | 0.2162 | -0.188 | 0.8521 |
| Trial | - 3.711 | 1.843 | -2.014 | 0.0447 |
| **Condition:** |  |  |  |  |
| Gesture congruent vs. Gesture incongruent | -106.6 | 51.42 | -2.073 | 0.0450 |

1. Experiment 1, Summary of mixed effects model 2 (comparison between gesture (in)congruent and no gesture conditions)

*rt.lmer = lmer(RT~condition+Onset_RE2_Onset_target_word+trial+(1|Subject),data)*

Random Effects

| **Group** | **Variance** | ***SD*** |
| --- | --- | --- |
| Subjects | 27260 | 165.1 |

Fixed Effects

| **Variable** | **Coeff. Estimate** | ***SE*** | ***t*** | ***p*** |
| --- | --- | --- | --- | --- |
| RE2 – target word | 0.01999 | 0.10174 | 0.196 | 0.844307 |
| Trial | -4.86310 | 1.49886 | -3.245 | 0.001238 |
| **Condition:** |  |  |  |  |
| Speech vs. Gesture congruent | 62.33991 | 37.63903 | 1.656 | 0.098158 |
| Speech vs. Gesture incongruent | -48.64506 | 33.45167 | -1.454 | 0.146383 |

1. Experiment 1, Additional analysis 2 (comparison between gesture (in)congruent and no gesture conditions, including ‘item’ as a random factor)

*rt.lmer = lmer(RT~condition+Onset_RE2_Onset_target_word+trial+(1|Subject)+ (1|Item),data)*

Random Effects

| **Group** | **Variance** | ***SD*** |
| --- | --- | --- |
| Subjects | 27230 | 165.02 |
| Item | 5632 | 75.05 |

Fixed Effects

| **Variable** | **Coeff. Estimate** | ***SE*** | ***t*** | ***p*** |
| --- | --- | --- | --- | --- |
| RE2 – target word | 0.01993 | 0.12621 | 0.158 | 0.87514 |
| Trial | -4.78984 | 1.48365 | -3.228 | 0.00131 |
| **Condition:** |  |  |  |  |
| Speech vs. Gesture congruent | 60.69513 | 49.45948 | 1.227 | 0.22775 |
| Speech vs. Gesture incongruent | -50.33034 | 44.82848 | -1.123 | 0.26974 |

**B**

1. Experiment 2, Summary of mixed effects model 1

*rt.lmer = lmer(RT~condition+Onset_prep_onset_RE2+trial+(1|Subject),data)*

Random Effects

| **Group** | **Variance** | ***SD*** |
| --- | --- | --- |
| Subjects | 4758 | 68.98 |

Fixed Effects

| **Variable** | **Coeff. Estimate** | ***SE*** | ***t*** | ***p*** |
| --- | --- | --- | --- | --- |
| G prep – RE2 | -0.2158 | 0.1061 | -2.033 | 0.04256 |
| Trial | -4.4216 | 1.3199 | -3.350 | 0.00087 |
| **Condition:** |  |  |  |  |
| Gesture congruent vs. Gesture incongruent | -3.3575 | 26.8363 | -0.125 | 0.90049 |

1. Experiment 2, Additional analysis 1 (comparison between gesture congruent and gesture incongruent conditions, including ‘item’ as a random factor)

*rt.lmer = lmer(RT~condition +Onset_prep_onset_RE2+trial+(1|Subject)+ (1|Item),data)*

Random Effects

| **Group** | **Variance** | ***SD*** |
| --- | --- | --- |
| Subjects | 4758 | 68.98 |
| Item | 0.000 | 0.000 |

Fixed Effects

| **Variable** | **Coeff. Estimate** | ***SE*** | ***t*** | ***p*** |
| --- | --- | --- | --- | --- |
| G prep – RE2 | -0.2158 | 0.1061 | -2.033 | 0.042552 |
| Trial | -4.4216 | 1.3199 | -3.350 | 0.000869 |
| **Condition:** |  |  |  |  |
| Gesture congruent vs. Gesture incongruent | -3.3575 | 26.8363 | -0.125 | 0.900486 |

1. Experiment 2, Summary of mixed effects model 2 (comparison between gesture (in)congruent and no gesture conditions)

*rt.lmer = lmer(RT~condition +trial+(1|Subject),data)*

Random Effects

| **Group** | **Variance** | ***SD*** |
| --- | --- | --- |
| Subjects | 3448 | 58.72 |

Fixed Effects

| **Variable** | **Coeff. Estimate** | ***SE*** | ***t*** | ***p*** |
| --- | --- | --- | --- | --- |
| Trial | -4.391 | 1.088 | -4.037 | 0.0000598 |
| **Condition:** |  |  |  |  |
| Speech vs. Gesture congruent | -18.921 | 23.259 | -0.813 | 0.4162 |
| Speech vs. Gesture incongruent | -51.505 | 23.481 | -2.193 | 0.0286 |

1. Experiment 2, Additional analysis 2 (comparison between gesture (in)congruent and no gesture conditions, including ‘item’ as a random factor)

*rt.lmer = lmer(RT~condition+trial+(1|Subject)+(1|Item),data)*

Random Effects

| **Group** | **Variance** | ***SD*** |
| --- | --- | --- |
| Subjects | 3430.9 | 58.57 |
| Item | 230.6 | 15.19 |

Fixed Effects

| **Variable** | **Coeff. Estimate** | ***SE*** | ***t*** | ***p*** |
| --- | --- | --- | --- | --- |
| Trial | -4.391 | 1.088 | -4.038 | 0.0000598 |
| **Condition:** |  |  |  |  |
| Speech vs. Gesture congruent | -19.063 | 23.965 | -0.795 | 0.4318 |
| Speech vs. Gesture incongruent | -51.721 | 24.180 | -2.139 | 0.0394 |
