# Supplementary material for: Addressees Are Sensitive to the Presence of Gesture When Tracking a Single Referent in Discourse
Source: Front Psychol. 2019 Aug 13;10:1775. doi: 10.3389/fpsyg.2019.01775 (PMC6700288; doi:10.3389/fpsyg.2019.01775)
Supplement: Supplementary file 2 [file Table_2.DOCX]

Stimulus sentences in German original version

1. *Da war eine Frau. Und ihr Sohn konnte seine Matheaufgaben nicht allein lösen. Also hat sich die Frau dazu entschlossen, eine Freundin anzurufen. Sie soll ihm dann zur Hilfe kommen.*
2. *Da war eine Frau. Und ihr Sohn konnte seine Matheaufgaben nicht allein lösen. Also hat sich die Frau dazu entschlossen, eine Freundin anzuschreiben. Sie soll ihm dann zur Hilfe kommen.*
3. *Da war ein Mädchen. Und ihr Freund konnte seine ganzen Kisten nicht allein in den Keller tragen. Also hat sich das Mädchen dazu entschlossen, ihren Vater anzurufen. Der soll ihm dann zu Hilfe kommen.*
4. *Da war ein Mädchen. Und ihr Freund konnte seine ganzen Kisten nicht allein in den Keller tragen. Also hat sich das Mädchen dazu entschlossen, ihren Vater anzuschreiben. Der soll ihm dann zur Hilfe kommen.*
5. *Da war ein Mädchen. Und ihr Vater konnte den Abfluss nicht selbst reparieren. Also hat sich das Mädchen dazu entschlossen, ihren Freund anzurufen. Der soll ihm dann zur Hilfe kommen.*
6. *Da war ein Mädchen. Und ihr Vater konnte den Abfluss nicht selbst reparieren. Also hat sich das Mädchen dazu entschlossen, ihren Freund anzuschreiben. Der soll ihm dann zur Hilfe kommen.*
7. *Da war ein Mädchen. Und ihre Mutter konnte die Kaffeemaschine nicht selbst reparieren. Also hat sich das Mädchen dazu entschlossen, ihren Vater anzurufen. Der soll ihr dann zur Hilfe kommen.*
8. *Da war ein Mädchen. Und ihre Mutter konnte die Kaffeemaschine nicht selbst reparieren. Also hat sich das Mädchen dazu entschlossen, ihren Vater anzuschreiben. Der soll ihr dann zur Hilfe kommen.*
9. *Da war ein Mädchen. Und ihre Freundin konnte ihr Bücherregal nicht allein zusammenbauen. Also hat sich das Mädchen dazu entschlossen, ihren Vater anzurufen. Der soll ihr dann zur Hilfe kommen.*
10. *Da war ein Mädchen. Und ihre Freundin konnte ihr Bücherregal nicht allein zusammenbauen. Also hat sich das Mädchen dazu entschlossen, ihren Vater anzuschreiben. Der soll ihr dann zur Hilfe kommen.*
11. *Da war eine Frau. Und ihr Sohn konnte den Kühlschrank nicht selbst reparieren. Also hat sich die Frau dazu entschlossen, seinen Vater anzurufen. Der soll ihm dann zur Hilfe kommen.*
12. *Da war eine Frau. Und ihr Sohn konnte den Kühlschrank nicht selbst reparieren. Also hat sich die Frau dazu entschlossen, seinen Vater anzuschreiben. Der soll ihm dann zur Hilfe kommen.*
13. *Da war eine Frau. Und ihre Tochter konnte ihren Laptop nicht mehr einschalten. Also hat sich die Frau dazu entschlossen, ihren Bruder anzurufen. Der soll ihr dann zur Hilfe kommen.*
14. *Da war eine Frau. Und ihre Tochter konnte ihren Laptop nicht mehr einschalten. Also hat sich die Frau dazu entschlossen, ihren Bruder anzuschreiben. Der soll ihr dann zur Hilfe kommen.*
15. *Da war eine Frau. Und ihre Mutter konnte ihre Spülmaschine nicht selbst reparieren. Also hat sich die Frau dazu entschlossen, den Handwerker anzurufen. Der soll ihr dann zur Hilfe kommen.*
16. *Da war eine Frau. Und ihre Mutter konnte ihre Spülmaschine nicht selbst reparieren. Also hat sich die Frau dazu entschlossen, den Handwerker anzuschreiben. Der soll ihr dann zur Hilfe kommen.*
17. *Da war eine Frau. Und ihr Mann konnte den Motor in seinem Auto nicht allein reparieren. Also hat sich die Frau dazu entschlossen, ihren Bruder anzurufen. Der soll ihm dann zur Hilfe kommen.*
18. *Da war eine Frau. Und ihr Mann konnte den Motor in seinem Auto nicht allein reparieren. Also hat sich die Frau dazu entschlossen, ihren Bruder anzuschreiben. Der soll ihm dann zur Hilfe kommen.*
19. *Da war ein Mädchen. Und ihr Freund konnte sein Motorrad nicht starten. Also hat sich das Mädchen dazu entschlossen, ihren Vater anzurufen. Der soll ihm dann zur Hilfe kommen.*
20. *Da war ein Mädchen. Und ihr Freund konnte sein Motorrad nicht starten. Also hat sich das Mädchen dazu entschlossen, ihren Vater anzuschreiben. Der soll ihm dann zur Hilfe kommen.*
21. *Da war ein Junge. Und seine Mutter konnte ihr Fahrrad nicht allein reparieren. Also hat sich der Junge dazu entschlossen, seinen Vater anzurufen. Der soll ihr dann zur Hilfe kommen.*
22. *Da war ein Junge. Und seine Mutter konnte ihr Fahrrad nicht allein reparieren. Also sich der Junge dazu entschlossen, seinen Vater anzuschreiben. Der soll ihr dann zur Hilfe kommen.*
23. *Da war ein Mann. Und seine Tochter konnte ihre Katze nicht allein zum Tierarzt bringen. Also hat sich der Mann dazu entschlossen, ihre Mutter anzurufen. Sie soll ihr dann zur Hilfe kommen.*
24. *Da war ein Mann. Und seine Tochter konnte ihre Katze nicht allein zum Tierarzt bringen. Also hat sich der Mann dazu entschlossen, ihre Mutter anzuschreiben. Sie soll ihr dann zur Hilfe kommen.*
25. *Da war ein Junge. Und seine Schwester konnte ihre Hose allein nicht richtig bügeln. Also hat sich der Junge dazu entschlossen, seine Mutter anzurufen. Sie soll ihr dann zu Hilfe kommen.*
26. *Da war ein Junge. Und seine Schwester konnte ihre Hose allein nicht richtig bügeln. Also hat sich der Junge dazu entschlossen, seine Mutter anzuschreiben. Sie soll ihr dann zur Hilfe kommen.*
27. *Da war ein Mann. Und seine Frau konnte das Abendessen nicht allein vorbereiten. Also hat sich der Mann dazu entschlossen, seine Tochter anzurufen. Sie soll ihr dann zur Hilfe kommen.*
28. *Da war ein Mann. Und seine Frau konnte das Abendessen nicht allein vorbereiten. Also hat sich der Mann dazu entschlossen, seine Tochter anzuschreiben. Sie soll ihr dann zur Hilfe kommen.*
29. *Da war ein Junge. Und seine Mutter konnte den Drucker nicht richtig bedienen. Also hat sich der Junge dazu entschlossen, seine Schwester anzurufen. Sie soll ihr dann zur Hilfe kommen.*
30. *Da war ein Junge. Und seine Mutter konnte den Drucker nicht richtig bedienen. Also hat sich der Junge dazu entschlossen, seine Schwester anzuschreiben. Sie soll ihr dann zur Hilfe kommen.*

English translation

1-2 ‘There was a woman. And her son could not solve his math homework by himself. So, the woman decided to call/write to her friend. She would come to help him out.’

3-4 ‘There was a girl. And her (boy)friend could not transport all his (moving) boxes into the basement by himself. So, the girl decided to call/write to her father. He would come to help him out.’

5-6 ‘There was a girl. And her father could not repair the leakage by himself. So, the girl decided to call/write to her (boy)friend. He would come to help him out.’

7-8 ‘There was a girl. And her mother could not repair the coffee machine by herself. So, the girl decided to call/write to her father. He would come to help her out.’

9-10 ‘There was a girl. And her friend could not assemble her bookshelf by herself. So, the girl decided to call/write to her father. He would come to help her out.

11-12 ‘There was a woman. And her son could not repair the refrigerator by himself. So, the woman decided to call/write to his father. He would come to help him out.

13-14 ‘There was a woman. And her daughter could not turn on her laptop anymore. So, the woman decided to call/write to her brother. He would come to help her out.’

15-16 ‘There was a woman. And her mother could not repair her dishwasher by herself. So, the woman decided to call/write to a handyman. He would come to help her out.

17-18 ‘There was a woman. And her husband could not repair on the engine of his car by himself. So, the woman decided to call/write to her brother. He would come to help him out.

19-20 ‘There was a girl. And her (boy)friend could not start his motorcycle. So, the girl decided to call/write to her father. He would come to help him out.’

21-22 ‘There was a boy. And his mother could not repair her bike by herself. So, the boy decided to call/write to his father. He would come to help her out.

23-24 ‘There was a man. And his daughter could not bring her cat to the vet by herself. So, the man decided to call/write to her mother. She would come to help her out.’

25-26 ‘There was a boy. And his sister was not able to iron her pants correctly. So, the boy decided to call/write to his mother. She would come to help her out.’

27-28 ‘There was a man. And his wife could not prepare the dinner by herself. So, the man decided to call/write to his daughter. She would come to help her out.’

29-30 ‘There was a boy. And his mother could not operate the printer correctly. So, the boy decided to call/write to his sister. She would come to help her out.’
