# Supplementary material for: Addressees Are Sensitive to the Presence of Gesture When Tracking a Single Referent in Discourse
Source: Front Psychol. 2019 Aug 13;10:1775. doi: 10.3389/fpsyg.2019.01775 (PMC6700288; doi:10.3389/fpsyg.2019.01775)
Supplement: Supplementary file 3 [file Table_3.docx]

### Consent form 1

### German original

*Einverständniserklärung*

*Ich gebe hiermit Sandra Debreslioska, Lund Universität, die Genehmigung die heutigen Aufnahmen (d.h., Antworten per Tastatur) für wissenschaftliche Analysen zu Forschungszwecken zu nutzen. Das bedeutet auch, dass die Ergebnisse der Analysen in Form von Graphen (oder ähnlichem) bei wissenschaftlichen Konferenzen, in Vorlesungen und in wissenschaftlichen Veröffentlichungen gezeigt werden.*

*Meine Anonymität ist voll und ganz garantiert. Unter keinen Umständen wird meine Identität offengelegt werden. Niemand außer der oben genannten Forscherin, wird über meine Identität Bescheid wissen (d.h., zum Beispiel, dass keine Namen in Präsentationen der Ergebnisse benutzt werden).*

### English translation

Consent form

Herewith I give Sandra Debreslioska, Lund University, the permission to use today’s recordings (i.e., key press answers) for scientific analyses for research purposes. This also means that the analyses/results will be shown in the form of graphs (or similar) at scientific conferences, in lectures or in scientific publications.

My anonymity is guaranteed. Under no circumstances will my identity be disclosed to anyone else than the above-mentioned researcher (e.g., no names will be used in presentations about the results).
