# Supplementary material for: Addressees Are Sensitive to the Presence of Gesture When Tracking a Single Referent in Discourse
Source: Front Psychol. 2019 Aug 13;10:1775. doi: 10.3389/fpsyg.2019.01775 (PMC6700288; doi:10.3389/fpsyg.2019.01775)
Supplement: Supplementary file 4 [file Table_4.docx]

### Consent form 2

### German original

*Einverständniserklärung*

*Ich gebe hiermit Sandra Debreslioska, Lund Universität, die Genehmigung die heutigen Aufnahmen (Audio und Video) für die folgenden Zwecke zu nutzen:*

*(Bitte, kreuzen Sie die entsprechende Box an,“", wenn Sie die Genehmigung dafür erteilen.)*

*1. Für wissenschaftliche Analysen zu Forschungszwecken;*

*2. Als Illustrationen der Analysen/Ergebnisse bei wissenschaftlichen Konferenzen, Vorlesungen und wissenschaftlichen Veröffentlichungen;*

*3. Als Illustrationen auf der Webseite der Universität Lund.*

*Meine Anonymität ist voll und ganz garantiert. Unter keinen Umständen wird meine Identität offengelegt werden. Niemand, außer der oben genannten Forscherin, wird über meine Identität Bescheid wissen (d.h., zum Beispiel, dass keine Namen in Präsentationen der Ergebnisse benutzt werden).*

### English translation

Consent form

I herewith give Sandra Debreslioska, Lund University, the permission to use today’s recordings (audio and video) for the following purposes:

(Please, cross the box, ‘’, if you give permission.)

1. For scientific analyses for research purposes;

2. As illustrations of the analyses/results at scientific conferences, lectures or in scientific publications;

3. As illustrations on Lund University’s website.

My anonymity is guaranteed. Under no circumstances will my identity be disclosed to anyone else than the above-mentioned researcher (e.g., no names will be used in presentations about the results or recordings).
